# Immunomodulation Pathogenesis and Treatment of Bone Nonunion

**DOI:** 10.1111/os.14131

**Published:** 2024-06-30

**Authors:** Chao Song, Yong Liu, Xingxing Tao, Kang Cheng, Weiye Cai, Daqian Zhou, Yang Zhou, Liquan Wang, Houyin Shi, Qi Hao, Zongchao Liu

**Affiliations:** ^1^ Department of Orthopedics and Traumatology (Trauma and Bone‐Setting), Laboratory of Integrated Chinese and Western Medicine for Orthopedic and Traumatic Diseases Prevention and Treatment, The Affiliated Traditional Chinese Medicine Hospital Southwest Medical University Luzhou China; ^2^ Department of Bone and Joint Sports Medicine Xingguo People's Hospital, Gannan Medical College Xingguo China; ^3^ College of Integrative Chinese and Western Medicine, The Affiliated Traditional Chinese Medicine Hospital Southwest Medical University Luzhou China; ^4^ Orthopedic Surgery, The Affiliated Traditional Chinese Medicine Hospital Southwest Medical University Luzhou China; ^5^ Department of Orthopedics Luzhou Longmatan District People's Hospital Luzhou China

**Keywords:** Bone Nonunion, Immunomodulation, Inflammatory Response, macrophages, T Cells

## Abstract

Fractures and bone nonunion commonly require surgical intervention. Serious outcomes of non‐healing in the late stages of fracture place a significant financial burden on society and families. Bone nonunion occurs when a fracture stops healing, for many reasons, and leads to a variety of bad outcomes. Numerous factors, including biomechanics and immunology, are involved in the complicated mechanisms of bone nonunion. The immune‐inflammatory response plays a significant part in the emergence of bone nonunion, and the occurrence, control, and remission of inflammation in the bone healing process have a significant influence on the ultimate success of bone tissue repair. In the bone microenvironment, immune cells and associated cytokines control bone repair, which is significantly influenced by macrophages, T cells, and fibroblast growth factor. To limit acute inflammation and  balance osteogenesis and osteoblastogenesis for tissue repair and regeneration, immune cells and various cytokines in the local microenvironment must be precisely regulated. As a bad complication of late‐stage fractures, bone nonunion has a significant effect on patients’ quality of life and socioeconomic development. Therefore, in‐depth research on its pathogenesis and treatment methods has important clinical value. To provide more precise, focused therapeutic options for the treatment of bone nonunion, we discuss the regulatory roles of the key immune cells engaged in bone healing within the microenvironment during bone healing and their effect on osteogenesis.

## Graphical abstract

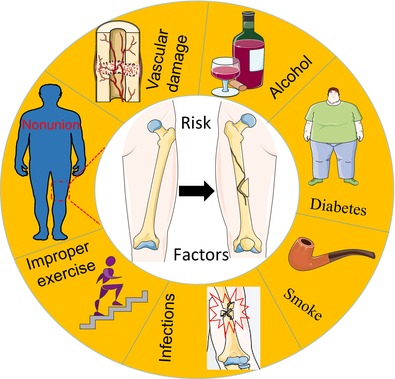

## Introduction

Fractures are the most common surgical trauma, with 5%–10% of patients experiencing delayed healing or bone nonunion, and bone nonunion is the most severe consequence of late fractures.^1^ Bone nonunio is defined by the U.S. Food and Drug Administration as “a condition that occurs at least 9 months after an injury or fracture and has not had a tendency to heal further for 3 months.” It occurs when a fracture stops healing for a variety of reasons and with a variety of complications.^1^, ^2^ Atrophic bone nonunion and hypertrophic bone nonunion are the two kinds of bone nonunion that are currently recognized. On radiographs, hypertrophic bone nonunion is seen as a massive bone crust at the fracture site and a fracture gap with widening healing tissue. Although hypertrophic bone nonunion has great potential for regeneration, it can be challenging to repair because of factors such as a lack of mechanical stability. Atrophic bone nonunion is distinguished by the absence of bone scab tissue, narrowing of the bone ends, and a significant radiographically permeable area in the fracture space. Further, there is no sufficient osteogenic response, no vascularity, and no indication of the creation of healing tissue or healing in atrophic osteogenesis.^3^, ^4^ Surgery, drugs, physical therapy, molecular biological therapy, and traditional Chinese medicine are used to treat bone fractures or bone nonunion. Specifically, bone nonunion requires extensive surgical therapy that often involves at least two operations. Inappropriate or delayed treatment might result in lifelong limb impairment, placing a significant psychological and financial strain on the patient.^5^ The prevalence and unfavorable prognosis of bone nonunion has become a serious issue for orthopaedic surgeons and places significant financial burden on society and families. Therefore, understanding the etiology and molecular mechanism of bone nonunion can assist in improving treatment plans and provide hope for better outcomes for individuals with the condition.

**FIGURE 1:**
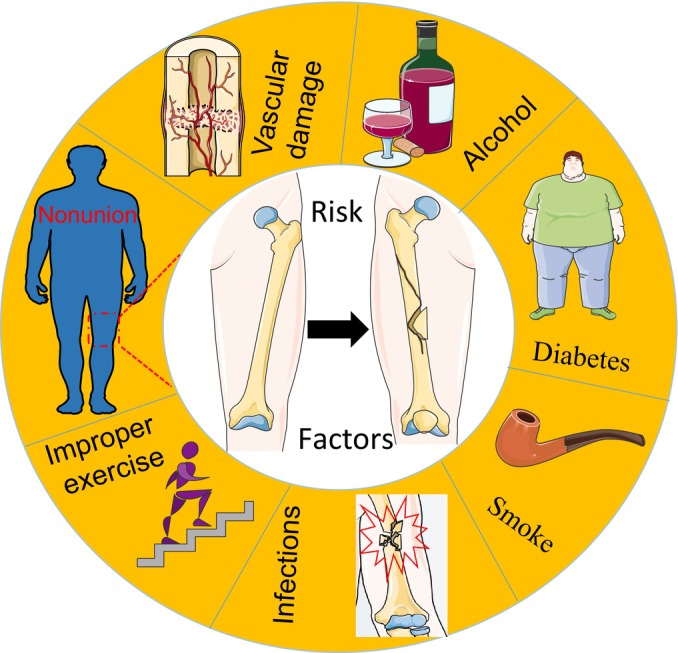
Common risk factors for bone nonunion: The risk factors for bone nonunion can generally be categorized into systemic and local factors. Systemic factors include obesity, diabetes mellitus, smoking, and alcohol consumption. Local factors are mainly caused by changes in the local environment of the bone nonunion, which mainly include local inflammation, local fixation instability, and local vascular injury.

One of the frequent side effects of bone fractures is bone nonunion, which has both systemic and local causes. Systemic causes of bone nonunion include diabetes mellitus, metabolic diseases, smoking, and drinking, as well as other systemic diseases that affect bone healing.^6^ Local factors include infection, mechanical instability, and lack of blood supply to the fracture area (Figure 1). The pathogenesis of bone nonunion is complicated, combining biomechanics, cell biology, immunology, anatomy, histology, and other domains, and a thorough understanding of its pathophysiology aids in the creation of clinical treatment strategies.^7^ Currently, there is considerable study being conducted on the role of immunoinflammatory variables in the development of bone nonunion.^8^ Additionally, earlier research revealed that fibroblasts’ aberrant expression and associated variables might be crucial in the development of bone nonunion.^9^ This article will explore in detail the pathogenesis of bone nonunion, especially the role of immune regulation, and discuss current treatment plans based on these mechanisms.

## Methods

**FIGURE 2:**
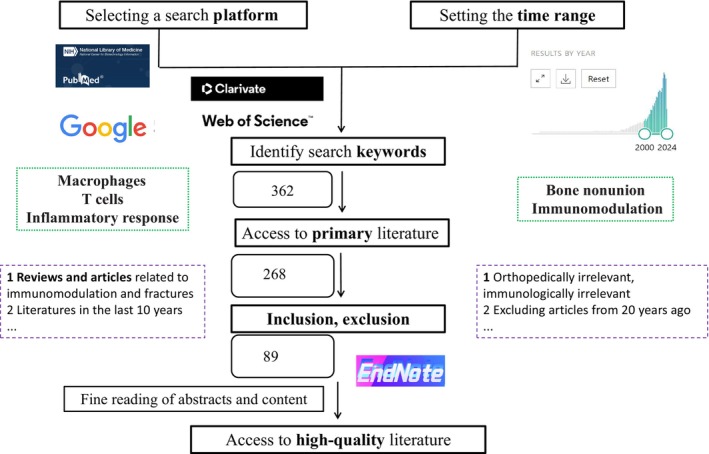
Flow chart: Article flowchart.

We used PubMed, Google Scholar, and Web of Science database search platforms, and the search time frame was 2000 to 2024. The search keywords, “bone nonunion,” “immunomodulation,” “macrophages,” “T cells” and “inflammatory response” were entered. The initial cataloging of articles, reviews, and treatises related to immune modulation and fracture was further clarified to prioritize the inclusion of articles published in the past 5 years. Articles not related to orthopaedics, not related to immunity, and published more than 20 years ago were excluded. Initially, 362 articles were obtained from the literature, again by reading the abstracts of the articles and excluding articles that were not relevant to the topic. A total of 268 high‐quality, relevant studies were obtained, and after intensively reading the full texts of these published studies, it was determined that the immune regulatory mechanisms of osteogenesis imperfecta were of research interest. A brief flow chart is presented in Figure 2.

### Biological Processes of Fracture Repair

**FIGURE 3:**
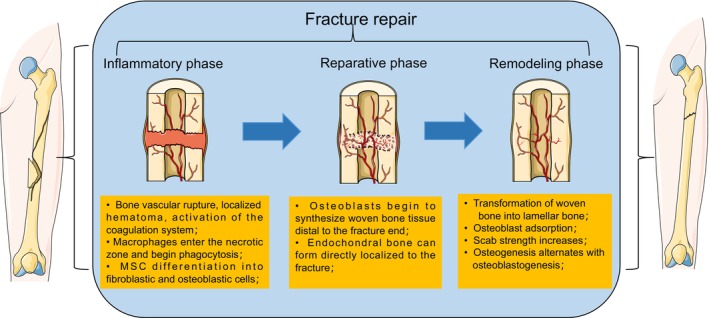
General stages of fracture healing: Healing after fracture is broadly divided into three stages. In the inflammatory phase, platelets release vasoactive mediators and inflammatory factors after the formation of a local hematoma. Macrophages phagocytose necrotic tissues and release signaling factors to promote the recruitment, migration, and proliferation of mesenchymal stem cells. In the proliferation stage, bone scabs are formed, and osteoblasts begin to synthesize woven bone tissue; in the remodeling stage, further bone scabs are formed, and mineralization and structural remodeling occur.

Anabolic and catabolic mechanisms are present throughout various stages of fracture healing.^10^, ^11^ The fundamental function of the severe inflammatory response that characterizes the early stages of healing is to clean the fracture site and support the signaling environment that propagates subsequent stages of healing, including the recruitment and differentiation of skeletal tissue progenitor cells.^12^ Following the inflammatory reaction, progenitor cells produce soft healing tissue, central cartilaginous areas visible, and peripheral new bone is created.^13^ Cartilaginous tissue characterized by avascular cartilage stimulates blood vessel formation during the anabolic phase.^13^ Endochondral ossification causes the beginning of the formation of hard bone tissue. Mineralization also increases and chondrocytes are partially replaced by osteoblasts through transdifferentiation.^14^ Finally, the healing tissue remodels through catabolic processes. The healing tissue shrinks, and osteoblastic and osteoclastic processes alternately rebuild normal hematopoietic and trabecular structures, returning the bone to its pre‐injury state.^15^ There are two types of fracture healing: direct healing and indirect healing. Direct healing requires artificial reconstruction of the bone cortex, anatomical realignment, and stabilization of the fracture ends to reestablish the Haversian system. An intricate process of bone repair known as indirect healing includes damage, inflammation, scab development, scab mineralization, and remodeling. An inflammatory phase, a proliferative phase, and a remodeling phase might typically be distinguished based on how the bone heals following a fracture (Figure 3).

#### Inflammatory Phase of Reparative Phase

After a fracture, the coagulation system is triggered, and degranulated platelets in the hematoma produce vasoactive mediators, which causes local bleeding from ruptured bone vessels and periosteal vessels.^16^ Several days after the injury, levels of numerous other inflammatory mediators—including IL‐1, IL‐6, IL‐18, and TNF‐alpha—were also noticeably increased.^17^ Following vascular injury, the local hypoxic environment prevents bone cells from receiving enough nutrition, which causes them to slowly deteriorate and necrose. The body then releases signaling substances, including bone morphogenetic protein 2 (BMP‐2), basic fibroblast growth factor, transforming growth factor (TGF), platelet‐derived growth factor (PDGF), and insulin‐like growth factor (IGF), to start the regeneration phase once macrophages enter the necrotic area and begin phagocytosis.^18^, ^19^ The recruitment, migration, and proliferation of MSCs as well as their differentiation into angiogenic, chondrogenic, fibroblastic, and osteoblastic cells are all facilitated by these cytokines.^19^ Fibroblasts and osteoblasts fill fracture gaps by forming granulation tissue.^20^ Normally, the inflammatory phase lasts up to a week, when the initial bone scab has gradually been produced.

#### Reparative Phase of Fracture

In this phase, which is characterized by the creation of bone crusts, the expansion of blood vessels, the secretion of osteoid, and the synthesis of collagen fibers, osteoblasts in the circulation and mononuclear precursor cells in the nearby bone marrow start to resorb the necrotic bone.^21^ Periosteal reaction, angiogenesis, connective tissue, and fibrous bone scab formation also occur at this stage and are eventually replaced by immature woven bone. MSCs differentiate into chondrocytes in a low oxygen environment, which develop into cartilage and support the fracture zone.^22^ The expression of several growth factors, including TGF‐2, PDGF, IGF, and BMP‐2, can promote the proliferation and differentiation of cartilage.^23^ While endochondral bone can grow directly localized to the fracture but is less stable, osteoblasts start to manufacture woven bone tissue distant to the fracture end. TGF‐2, BMPs, and other signals cause the cartilaginous scab to gradually calcify and eventually become woven bone.^24^

#### Remodeling Phase of Reparative Phase

The remodeling phase involves structural remodeling, which is often referred to as secondary bone development and involves the conversion of irregularly woven bone into lamellar bone, as well as the formation of bone crust and mineralization.^25^ Osteoblasts gradually resorb the newly formed woven bone during this phase and replace it with lamellar bone. Osteoclasts eventually polarize and cling to the surface as the remodeling process progresses. For osteoblasts to be deposited on the surface of the developing bone, they produce tight crease borders where proteases enter the resorption area and osteoclasts start to take up bone tissue in the traps.^25^ However, osteoclasts in the response process still differs in dense bone from cancellous bone. This ongoing process is somewhat comparable to the mechanism of substitution repair in normal bone. Cells in cancellous bone are found close to blood arteries, and a process known as “creeping substitution” causes bone to deposit on the trabecular bone's surface.^26^ The remodeling process is regulated by a number of pro‐inflammatory signals, such as IL‐1 and IL‐6 and elevated TNF‐α, IL‐12, and IFN‐γ.^27^ The healing process and scab strength are also accelerated and strengthened during this phase by growth hormone and parathyroid hormone. With positive charges on the bone surface when pressure is applied (linked to osteoblastic activity) and negative charges (associated with osteoclastic activity), the electrophysiology also affects bone remodeling, although the precise process remains unknown.^28^

### Immuno‐Inflammatory Mechanisms of Bone Nonunion

#### Bone Nonunion and Immunization

Clinically, bone nonunion is primarily associated with post‐traumatic infection and bone abnormalities, while other variables, including inadequate local blood supply, unsuitable internal fixation therapy, and frequent and improper manipulation and repositioning, can also raise the risk of bone nonunion.^22^ Bone nonunion can be broadly divided into hypertrophic bone nonunion, atrophic bone nonunion, infected bone nonunion, and aseptic bone nonunion based on clinical presentations, X‐ray manifestations, and whether or not infection is present.^29^ The process of fracture nonunion is complex, involving cell biology, immunology, anatomy, histology, and other domains, and the role of immunological factors in bone nonunion has been a focus of investigation.^30^ As a result, we outline the mechanism of the occurrence of immunity and bone nonunion by combining domestic and foreign research breakthroughs. Horton *et al*. revealed a putative regulatory link between the immune system and bone over 40 years ago.^31^ When leukocytes in the peripheral circulation become activated, they release a water‐soluble chemical that promotes osteogenesis. As cellular and molecular biology research has progressed, it has been discovered that this substance is a complex of multiple molecules; that is, multiple molecules are involved in the regulation of bone remodeling as well as the immune response, such as through the NF‐κB signaling pathway and the RANK signaling pathway.^32^ Recent investigations have revealed that the RANK signaling pathway also plays a significant regulatory role in osteoclast development in addition to being first identified as a regulator of T cell and dendritic cell function.^33^ In 2000, ARRON *et al*. introduced the concept of osteoimmunology, formally linking the immune system to bone. The relationship between osteoimmunology and the onset of osteoarthritis, lumbar spine degenerative lesions, and fracture healing has emerged as a popular research area.^34^, ^35^ Initially, it was believed that immunomodulation was only important in the early stages of fracture healing and that the middle and late stages of fracture healing were primarily influenced by mechanistic factors. However, more recent research has revealed that immunomodulation is crucial throughout the entire fracture healing process.

Disturbances in immunoregulation during the period of bone scab shaping can result in an imbalance in osteogenic‐osteoblastic homeostasis, which can lead to slow or terminated shaping. Immune cells are heavily involved in the bone‐mending process. Immune cells like neutrophils, macrophages, T cells, and natural killer (NK) cells are among the first inflammatory cells to reach the bone microenvironment after bone damage.^36^ Immune cells recruit bone marrow mesenchymal stem cells into the bone tissue and control their proliferation, differentiation, or apoptosis during the early stages of inflammation. These immune cells release a variety of cytokines and chemokines that entice different types of cells into the bone injury zone to participate in the inflammatory response.^36^, ^37^ Osteogenesis and osteoblastogenesis interact with immune cells through regulatory pathways common to both, such as nuclear factor κB receptor activating factor ligand/nuclear factor κB receptor activating factor/osteoprotegerin (RANKL/RANK/OPG) interactions, a phenomenon referred to as the osteoimmunomodulatory effect.^38^ The inclusion of an overview of bone's immunomodulatory effects places special emphasis on the host's immunological reaction to the graft during bone formation and the crucial regulatory role of immune cells during bone repair, fostering a favorable immune milieu for bone healing.

#### Bone Nonunion and Intrinsic Immunity

Intrinsic immunity, commonly referred to as non‐specific immunity, is an innate physiological defensive mechanism that includes, in particular, tissue barriers, intrinsic immune cells, and intrinsic immune chemicals.^34^ Dendritic cells, NK cells, mast cells, and other phagocytes (e.g., neutrophils, macrophages, and mononuclear phagocytes) are examples of intrinsic immune cells. Intrinsic immune molecules include the complement system, cytokines, and acute phase proteins.^34^ Hematoma formation is caused by the fracture's initial local vascular disruption. Neutrophils are concentrated in the fracture area for the first 24 hours and emit chemokines like CCR2 and IL‐6 to draw inflammatory cells such as monocytes and macrophages to the infiltrate.^39^ Through cytophagy, macrophages eliminate the original fibrous tissue and necrotic cells, while osteoclasts control the resorbing necrotic fracture fragments. Additionally, through secreting a range of inflammatory mediators and chemokines (e.g., TNF‐ α, IL‐1β, IL‐6, and MCP‐1), macrophages attract fibroblasts, osteoblast precursors, and mesenchymal stem cells (MSCs) locally in the bone marrow, periosteum, and capillaries.^39^ The hematoma gives way to granulation tissue, neovascularization, and sensory nerve fibers within a week, all of which are crucial for the healing of fractures.^40^ After that, cartilage scabs gradually develop in the fracture area, offering the essential support for the subsequent phase of bone remodeling. Due to their great consistency and adaptability during this process, macrophages play a crucial part in the immunomodulation of tissue remodeling.

Macrophages can be divided into pro‐inflammatory (M1‐type) and anti‐inflammatory (M2‐type) macrophages depending on how they are activated. Following a fracture, there is a fast inflammatory response dominated by M1‐type macrophages, followed by an osteoclast recruitment response dominated by M2‐type macrophages, and both M1 and M2 types have a pro‐osteogenic effect.^40^ Loi *et al*. found that co‐culturing macrophages with preosteoblasts significantly upregulated the expression levels of osteogenesis‐related genes such as alkaline phosphatase (ALP), osteocalcin (OC), and osteoblastin (OPN), as well as matrix mineralization.^41^ When exposed to IFN‐γ and lipopolysaccharide (LPS), aged mouse M1 macrophages’ mRNA expression and protein secretion of TNF‐α were both significantly upregulated, according to research by Gibon *et al*., but IL‐1 receptor antagonist (IL‐1RA) secretion was not affected by LPS exposure.^42^ *In vitro* experiments revealed that M1‐type macrophages were able to inhibit MSC activity, whereas M2‐type macrophages had the opposite effect.^43^ Numerous animal studies that impaired macrophage function have validated the significance of macrophages in fracture repair. On the periosteal surface of young rats, endosteal macrophages were discovered to create niches around osteoblasts. FAS triggered macrophage death, whereas knockout rat macrophages dramatically hindered osteoblast development.^44^ Using the same model, Vi L *et al*. found that early skeletal development was delayed in rats after macrophage knockout and that both the number of MSCs and their ability to differentiate into osteoblasts were significantly reduced.^45^ This demonstrates that macrophages are crucial to the early inflammatory phase of fracture healing as well as the middle phase of bone regeneration.

**FIGURE 4:**
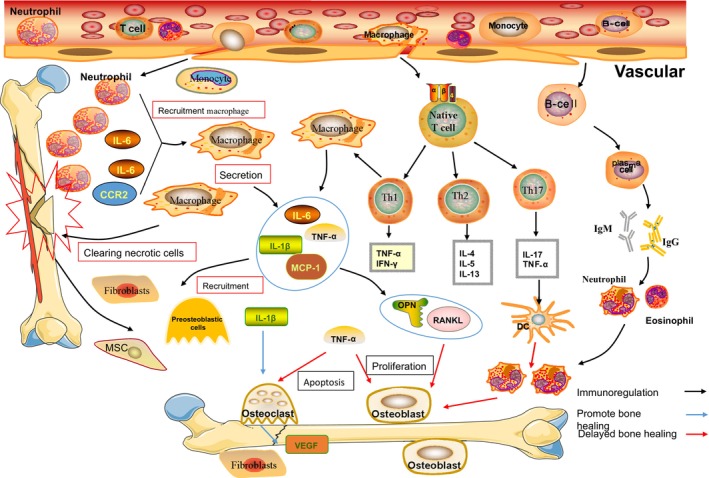
Immune mechanisms involved in the development of bone nonunion: (A) Post‐fracture release of neutrophils induces the aggregation of innate immune cells such as monocytes and macrophages, which again release a number of inflammatory mediators that are capable of recruiting fibroblasts, MSCs, and preosteoblasts for production and aggregation; IL‐1β mainly promotes the transformation of preosteoblasts into osteoblasts, and TNF‐α mainly promotes osteoclast differentiation and formation. Overexpression of factors related to osteoclast promotion will lead to bone dysplasia. In addition, T‐cells, in the inflammatory environment, differentiate into Th1, Th2, and Th17, of which IL‐17 and TNF‐ α will recruit dendritic cells, which will further lead to overaggregation of neutrophils, overactivation of osteoclasts, and an increase in bone destruction.

Macrophages are closely related to osteoclasts, and after fracture injury osteoclasts can be rapidly recognized by intrinsic immune cells, especially macrophages, and initiate a series of downstream cascade reactions to replace the hematoma with fibrous connective tissue.^46^ Mature macrophages are able to take part in osteogenic repair processes as osteoclast precursors and can be coaxed to develop into osteoclasts in a laboratory setting from a variety of origins of macrophages.^46^, ^47^ In addition, different types of external stimuli induce macrophages to secrete different cytokines, such as IL‐1, IL‐6, and TNF‐α, which stimulate/prevent osteoclasts from proliferating and differentiating, thereby regulating bone regeneration.^48^ TNF‐α and IL‐1 promote osteoclast differentiation and maturation through downregulation of osteoprotegerin in osteoblasts and promotion of nuclear factor‐κB receptor‐activating factor ligand (RANKL) expression, and they are able to exert synergistic effects.^49^ By causing osteoclast death, TNF‐α can stimulate osteoclast proliferation. While this is going on, TNF‐α changes M2‐type macrophages into M1‐type macrophages, and M1‐type macrophages can increase the potential for osteoclast production, which will increase osteoclast action.^50^ Some scholars have found that TNF‐α plays an important role in recruiting osteoclasts, MSCs, and inducing chondrocyte apoptosis by knocking down the TNF‐α receptor and that sustained high levels of TNF‐α expression lead to the development of chronic inflammation and bone loss, and even to the development of rheumatoid arthritis‐like symptoms, which slows down the healing of fractures.^51^ In contrast, IL‐1 stimulates osteoblasts to produce IL‐6, which promotes vascular endothelial growth factor (VEGF) production and participates in primary cartilage scabs and neovascularization in the fracture localization to promote bone repair.^52^ OPN has been demonstrated to be expressed in macrophages and to contribute to the chemotaxis of these cells toward inflamed areas.^53^ Mc Kee *et al*. found that OPN secreted by macrophages and osteoblasts after fracture has an important role in fracture healing: macrophages remove bone fragments by phagocytosis and form an adhesive line at the fracture site, thus integrating the repair of new bone.^53^ In addition, macrophages can participate in the composition of the osteogenic microenvironment and promote osteoblast differentiation and bone regeneration by regulating osteoblast matrix formation and mineralized deposition (Figure 4).

The association between macrophages and osteogenic healing is a novel topic of study, and in recent 10 years, much attention has been given to investigating the many types of macrophages and their roles in bone metabolism in various animal models. The method by which macrophages control bone metabolism and bone regeneration, however, is currently poorly understood, and this mechanism should be further studied in the future.

#### Bone Nonunion and Adaptive Immunity

**FIGURE 5:**
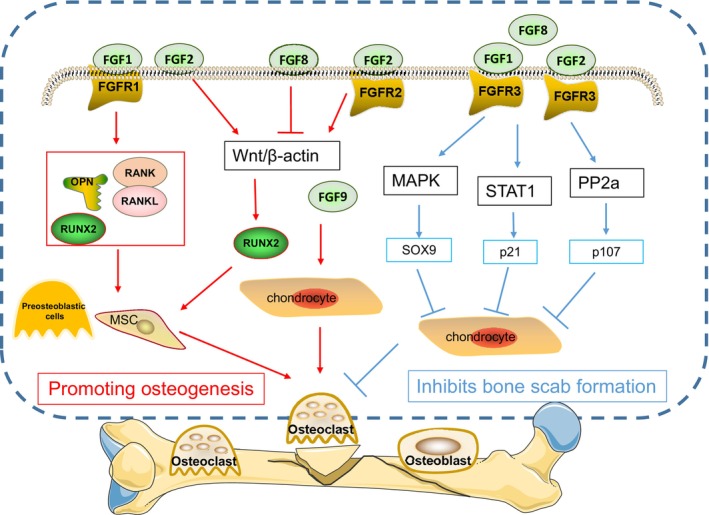
Mechanisms of fibroblasts and their factors in bone nonunion: FGF1 and FGF2 mainly promote the aggregation of mesenchymal stem cells (MSCs) and fibroblasts through osteogenesis‐related proteins to promote osteogenesis, and FGF8 inhibits Wnt/β‐actin signaling to inhibit osteogenesis; FGFR3 inhibits fibroblast generation through various signaling factors, which makes the osteoblasts and osteoclasts lose the adhesive structure, thus inhibiting bone healing.

Adaptive immunity, also known as specific immunity, differs from intrinsic immunity in that the cells that perform adaptive immunity include T cells and B cells.^34^, ^35^ Initially, it was thought that only intrinsic immunity played a role in fracture healing, while adaptive immunity only played a role in other diseases, such as infections. However, with further research, it was found that adaptive immunity and intrinsic immunity complemented and influenced each other, and decreased adaptive immunity could lead to slow fracture healing^32^, ^33^, ^54^ (Figure 5).

Mice lacking lymphocytes might show the opposite phenomenon of delayed or accelerated fracture healing, with different subpopulations of T cells performing their respective functions in bone repair and with CD8+ T cells delaying fracture healing.^55^ Activated T cells enhanced the expression of RANKL, IFN‐γ, TNF‐α and OPG, and RANKL induced osteoclast differentiation and promoted bone resorption *via* the RANK‐RANKL signaling pathway. IFN‐γ induces RUNX‐2 downregulation and promotes IFN‐γ‐mediated apoptosis in BMSC; TNF‐α inhibits NF‐κB signaling in BMSC and converts IFN‐γ‐activated nonapoptotic Fas into caspase‐8/3‐related apoptotic signaling, leading to BMSC.^56^, ^57^ T‐cell receptor TCR knockout mice showed significantly higher expression of type II collagen, BSP, and BMP‐2‐related osteogenic genes as well as significantly lower levels of the inflammatory cytokines IL‐2, IFN‐γ, and IL‐6 produced at the fracture site, indicating that the lack of T cells promotes bone healing.^58^ CD4+ T cells can differentiate into multiple subtypes of T cells, depending on the peptides presented to them by major histocompatibility complex (MHC) class II molecules on antigen‐presenting cells (APCs) into Th1, Th2, and Th17 subpopulations.^57^ Differentiation of Th1 cells is induced by IL‐2, IL‐12, and IFN‐γ. The main effector cytokines are IFN‐γ and TNF‐α. Th1 cells recruit macrophages and induce IgG production by B cells. Th2 cells are induced to differentiate by IL‐4, and the main effector cytokines are IL‐4, IL‐5, and IL‐13. Th2 cells recruit eosinophils, basophils, and mast cells and mediate the production of IgE and IgG by B cells. eosinophils, basophils and mast cells and mediate the production of IgE and IgG by B cells.^34^ Th17 cells are a unique type of T helper cell development. In the early stage of acute inflammation, Th17 cells can be recruited toward the bone‐healing region under the action of chemokines CCL2 and CCL20 released by neutrophils, and the large amount of recruited Th17 strengthens the acquired immune response of the organism. Activated Th17 cells express IL‐17 and activate DC cells to release G‐CSF, which induces a large number of neutrophils, resulting in a sustained inflammatory response, affecting BMSC recruitment and osteogenic differentiation and ultimately leading to delayed bone healing or injury.^34^, ^59^, ^60^ Dixit *et al*. used anti‐IL‐17 to treat osteoporosis in female mice and found that anti‐IL‐17 treatment inhibited osteoclast activity, promoting proliferation and differentiation of osteoblasts and reversing inflammatory responses due to immune cell senescence; it had a protective effect against bone loss in ovariectomized mice.^61^ Therefore, targeting of the inflammatory factor IL‐17 in the bone microenvironment could promote new bone regeneration, enhance the expression of osteogenic markers, and reduce oxidative stress at the site of injury. Although it has been proposed that Th17 cells produce IL‐17F to promote bone healing, no enhanced expression of IL‐17F was found in injured tissues.^62^ It is evident that the role of CD4+ T cells in fracture healing needs to be further investigated.

#### Bone Nonunion and Fibroblast Growth Factor

We now understand the connection between different immune responses and the emergence of bone nonunion, and we have also discovered that fibroblast growth factor (FGF) signaling is crucial for the formation of bones.^63^ There are 22 members of the FGFs family, which can be categorized into endocrine FGFs (FGF15/19, FGF21, FGF23), intracellular FGFs (FGF11–14), and classical FGFs. Its receptors, fibroblast growth factor receptors (FGFRs), belong to a family of tyrosine kinase‐type receptors, with four types, FGFR1 to 4.^64^ Classical FGFs bind to their relatively specific FGFRs with heparan sulfate (HS) or heparan sulfate acetylated proteoglycans (HSPG) as co‐receptors to form the FGF‐FGFR‐HS complex. This complex activates the structural domain of FGFR intracellular tyrosine kinase through the phosphorylation of specific tyrosine residues and further activates intracellular signaling pathways including RAS‐MAPK, PI3K‐AKT, PLCγ, and STAT, which are involved in the regulation of early embryonic development, organogenesis, maintenance of metabolism, and tissue repair and regeneration and play particularly important roles in the process of bone development.^64^, ^65^

Fibroblast growth factors might be involved in the regulation of bone damage repair, according to related genetic modification mouse and *in vitro* intervention investigations. FGF1 treatment promotes the proliferation of human osteogenic precursor mesenchymal stromal cells and induces the expression of osteoblast‐specific differentiation markers, whereas FGF1 might promote bone repair by inhibiting adipogenic differentiation, increasing the number of osteoblasts in the inflammatory milieu, and orchestrating bone formation and angiogenesis during the bone repair process.^66^, ^67^ By controlling chondrocyte and osteoblast differentiation and vascular infiltration, low molecular weight FGF2 overexpression in mouse osteogenic precursor cells speeds up the healing of tibial fractures. It also promotes the healing of cranial bone defects by enhancing traditional Wnt signaling and osteogenic activity.^68^ FGF6 therapy had an effect on both osteoblast and osteoclast functions, according to *in vitro* experiments, indicating that it might be regulating bone damage repair.^69^ By disrupting Wnt signaling, FGF8 enables cranial bone‐associated precursor cells to more easily develop into cartilage, indicating the FGF8 signaling pathway as a negative regulator of osteogenic destiny.^70^ The expression of FGF9 is necessary to change some mesenchymal cells’ differentiation fate from intramembranous to endochondral osteogenesis. Mature osteoblasts are an important source of FGF9, which might maintain osteoblastic progenitor cell function by activating the AKT signal. FGF9 promotes chondrocyte hypertrophy at an early stage and regulates vascularization and osteogenesis in the growth plate at a later stage of skeletal development.^71^, ^72^ In animal models, a variety of FGFs and FGFRs have been shown to have relatively specific expression during fracture repair. Earlier studies found that FGF1, 2, and 5 were significantly elevated during the post‐fracture inflammatory phase by quantitative PCR assay. During the cartilage formation stage, the expression of FGF16 and 18 peaked, while during the hard bone scab formation and remodeling stage, there was a high expression of FGF2, 9, 16, and 18, while the expression of FGF1 and 17 peaked.^73^

**FIGURE 6:**
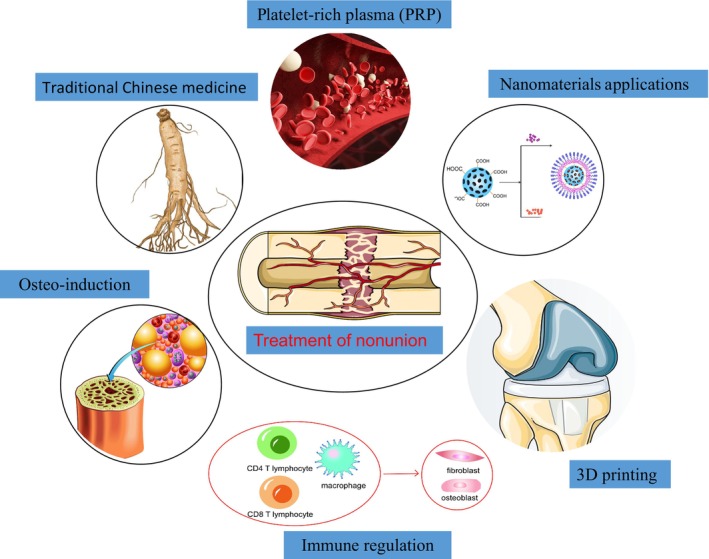
Some of the therapeutic options proposed to address the mechanisms of osteogenesis: platelet‐rich plasma, use of nanomaterials, three‐dimensional printing for bone defects, use of immunomodulators, osteoinductive factors, and treatment with traditional Chinese medicine.

FGFR1–3 are all engaged in the regulation of chondrogenesis and bone formation in addition to FGFs, which are also involved in bone healing. FGFR3 has a rather significant involvement in the process of chondrogenesis. It is generally acknowledged that FGFR3 inhibits chondrocyte proliferation and differentiation and increases chondrocyte apoptosis, whereas FGFR1 promotes osteoblast proliferation and differentiation and FGFR2 stimulates osteoblast proliferation, differentiation, and apoptosis. FGFRs regulate skeletal‐associated cell functions through their downstream signaling pathways.^74^ Genetic polymorphisms of FGFR1 are associated with fracture osteoarthritis.^74^ Through the overexpression of Wnt/β‐catenin, FGFR2 increases bone growth after mechanically removing the bone marrow from long bones, according to research using the FGFR2 gain‐of‐function point mutant mice model.^75^ Using FGFR3 function enhancement and an FGFR3 knockout mouse tibia fracture model, we found that FGFR3 inhibits cartilage scab formation and thus delays bone injury repair mainly through negative regulation of endochondral osteogenesis.^76^, ^77^ Knockdown of FGFR3 inhibits injured tissue remodeling after osteocortical injury *via* osteoclast bone resorption in a mouse osteoblast cell line.^78^ The fact that FGFRs are widely expressed in a variety of cell types during fracture healing, including mesenchymal cells, chondrocytes, osteoblasts, osteoclasts, and inflammatory cells, suggests that FGFRs signaling is involved in bone injury repair by regulating a variety of cellular profiles involved in fracture healing (Figure 6). However, more research is required to fully understand the specific functions and mechanisms of FGFRs (Table 1).

**TABLE 1:** Summary of the mechanisms of immune‐related cells and factors in fracture repair

Main immune cells	Effect on fracture repair
Neutrophils	Enriched to the fracture area and releases IL‐6, CCR2 etc. to recruit inflammatory cells, and mononuclear macrophages begin to infiltrate
Macrophages	1. Remove the initial fibrous tissue and necrotic cells
	2. Secrete a variety of inflammatory mediators and chemokines to recruit fibroblasts, osteoblast precursors, and mesenchymal stem cells
	3. Regulate osteoblast matrix formation and mineralization deposition, participate in the composition of the osteogenic microenvironment, and promote osteoblast differentiation and bone regeneration
M1 macrophages	Dominant rapid inflammatory response, inhibits MSC activity, and enhances osteoclast formation potential
M2 macrophages	Dominant osteoblast recruitment response
Th1	Recruit macrophages and induce B cells to produce IgG
Th2	1. Differentiation is induced by IL‐4, and its main effector cytokines are IL‐4, IL‐5, and IL‐13.
	2. Recruit eosinophils, basophils and mast cells and mediate B cells to produce IgE and IgG
Th17	1. Expression of IL‐17 activates D C cells to release G—C SF and induce the accumulation of a large number of neutrophils, causing a sustained inflammatory response
	2. Affect BMSC recruitment and osteogenic differentiation, leading to delayed bone healing or damage
FGF	1. FGF1 treatment promotes the proliferation of human osteoblast precursor mesenchymal cells and induces the expression of osteoblast‐specific differentiation markers
	2. FGF1 might promote bone repair by inhibiting adipogenic differentiation, increasing the number of osteoblasts in an inflammatory environment and coordinating bone formation and angiogenesis during bone repair
	3. FGF2 can accelerate the healing process of tibial fractures by regulating chondrocyte and osteoblast differentiation and vascular infiltration
	4. FGF6 treatment can affect the functions of osteoblasts and osteoclasts at the same time, suggesting that it mihgt be involved in the regulation of bone damage repair
	5. FGF8 causes skull‐related progenitor cells to be more susceptible to chondrogenic differentiation by causing dysregulation of Wnt signaling, suggesting that the FGF8 signaling pathway is a negative regulator of osteogenic fate
FGFRs	1. FGFR1 promotes osteoblast proliferation and differentiation
	2. FGFR2 stimulates osteoblast proliferation, differentiation, and apoptosis
	3. FGFR3 inhibits chondrocyte proliferation and differentiation, and promotes chondrocyte apoptosis

### Therapeutic Strategies for Bone Nonunion

Treatment options for fracture nonunion range from encouraging tissue differentiation to generate new bone to directing new bone to grow into the defect location, as well as a mix of therapeutic approaches, depending on the mechanism by which it promotes bone healing. Osteoinduction is the process by which a tissue or its extract stimulates the differentiation of primitive undifferentiated pluripotent stem cells into bone. Cytokines are important for initiating osteoblast differentiation and proliferation.^79^ When bones are mending, a class of small molecular weight proteins called cytokines, which can control cell function, is typically present in the periosteal matrix. Cytokines can control cell proliferation, differentiation, maturation, and other processes. Among these, BMP, VEGF, PDGF, and others are engaged in the repair and reconstruction of the bone tissues and can be used as a potential substitute in the treatment of subpar bone healing.^79^ A large number of animal experimental results have supported the application of the above cytokines for the treatment of osteogenesis imperfecta, but the progress of clinical application studies remains limited, considering the restrictions around safety, stability, and cost‐effectiveness. At present, only rhBMP‐7 and rhBMP‐2 have been approved by the FDA to form a commercialized product (approved in 2001 and 2002, respectively).^80^

In addition, platelet‐rich plasma (PRP) injection and autologous bone marrow injection are now commonly used clinically for the treatment of poor bone healing. PRP is a platelet‐rich high‐concentration plasma made from one's own blood, which includes active factors such as TGF‐β1, PDGF, PF4, IL‐1, IGF‐1, osteocalcin, osteoconjugate, fibronectin, and fibrinogen, which can promote the proliferation and differentiation of osteoblasts. In recent 10 years, several clinical trials have confirmed that local injection of PRP can help to improve the state of poor bone healing.^81^ However, using PRP alone does not speed up or accelerate fracture healing; instead, it works best when paired with bone grafting, for which additional research is still needed to determine the precise effectors and how they work.

Osteoconduction is the process by which new bone tissue grows on a material's surface or the surface of a pore, channel, or canal. The continuous existence of proliferating scar tissue in a non‐anatomically healed bone fracture region can hinder the normal division of the fracture ends, leading to bone nonunion. Surgical removal of the fracture scar is necessary before bone healing can take place. However, insufficient scar cleaning will result in limb shortening; therefore the defect space needs to be filled. The most fundamental criterion of a filler is that it must be able to ensure osteoconduction (i.e., act as a bridge to facilitate the formation of new blood vessels and bone). The optimal filler should contain osteoblasts, bone formation‐inducing agents, and other ingredients in addition to being a scaffold. The fillers commonly used today are autogenous, allograft, xenograft, and artificial bone.^82^ Autologous bone graft is the gold standard of bone grafting.^83^ The method of iliac bone extraction is recommended to minimize the discomfort to the patient by using the open window method of iliac bone extraction. Allogeneic bone refers to bone tissue of the same species but is genetically different from that in other individuals and is considered the best alternative to autologous bone grafting. Allogeneic bone grafting is also a commonly used bone grafting modality in clinical practice, with a very high annual average usage. Allograft bone itself has no osteoinductive effect, and its promotion of bone healing mainly relies on osteoconduction, so it is more often used in filling bone defects.^84^ As an alternative to autologous bone grafting, the strategy of co‐transplanting allogeneic bone compounded with BMP and angiogenic factors has been shown to be effective, and it is also a potential direction for future clinical promotion and application. VEGF‐treated allogeneic bone has better osseointegration results.^85^

In addition to the traditional treatments mentioned above, in recent 5 years, treatment with traditional Chinese medicine and cytokine‐targeted therapy based on immune modulation have also gradually played an important role. Resveratrol activates SIRT1 and improves lipid metabolism in the form of regulation of PPAR‐γ downregulation of PPAR‐γ expression and enhanced osteogenesis. Resveratrol promotes bone repair in MSCs through Wnt/β‐catenin.^86^, ^87^ Fibroblast growth factor is an effective cell‐dividing factor that acts on mesenchymal and neural ectodermal origin cells. It also plays a role in the differentiation of a wide variety of cells and is essential for morphogenesis, angiogenesis, and development. FGF has been demonstrated to support bone regeneration, but it has a short *in vivo* half‐life; thus, it must be combined with a proper carrier delivery to extend the time it is active. FGF has also been linked to bone regeneration. In addition, the frequency of drug delivery is an important factor; it has been shown that continuous treatment with FGF2/9 inhibits osteogenesis and mineralization of osteoblasts, whereas brief pretreatment significantly enhances mineralization.^88^ Because FGFs/FGFRs play a significant role in bone healing and regeneration, controlling FGF signaling might help fracture healing. Currently, our understanding of the complex role of FGF signaling and its mechanism in fracture healing is very limited. realizing clinical translational uses for bone regeneration (Table 2).

**TABLE 2:** Summary of main treatment strategies for nonunions.

Main treatment strategies	Mechanism
Cytokines	Bone morphogenetic protein (BMP), vascular endothelial growth factor (VEGF), and platelet‐derived growth factor (PDGF) are involved in the repair and reconstruction process of bone tissue.
Platelet rich plasma	Active factors such as TGF‐β1, PDGF, PF4, IL‐1, IGF‐1, osteocalcin, osteonectin, fibronectin, and fibrinogen promote the proliferation and differentiation of osteoblasts.
Material	Autologous bone, allograft bone, xenograft bone and artificial bone. The gold standard for autologous bone transplantation is still the iliac bone.
Fibroblast growth factor	A potent mitogen for cells of mesenchymal and neuroectodermal origin; it also plays a role in the differentiation of a variety of cells and participates in morphogenesis, angiogenesis, and development.
Traditional Chinese medicine	Resveratrol can activate SIRT1 and improve lipid metabolism by regulating PPAR‐γ. The expression of PPAR‐γ is downregulated and osteogenesis is enhanced. Resveratrol promotes mesenchymal stem cell bone repair through Wnt/β‐catenin.

## Discussion

According to the conventional theory, fracture healing is separated into three phases: the hematoma‐inflammatory mechanization period, the scab creation phase, and the scab shape phase. However, this staging does not completely explain the process of fracture healing.^10^, ^11^ The relationship between immune cells and fracture healing was investigated by Baht *et al*. in 2018. They divided fracture healing into four stages, including the inflammatory response stage, cartilaginous bone scab formation stage, osseous bone scab formation stage, and bone scab shaping stage, and came to the conclusion that different immune cells intervene in each stage, interact with osteoclasts and osteoblasts, and secrete cytokines, vesicles, and other substances.^89^ At the same time, osteoblasts and osteoclasts also secrete factors that counteract the immune cells, and the cellular phenotype and cytokines secreted by the same immune cells vary at different stages of fracture healing.^89^ In conclusion, as research has progressed, our understanding of how bones heal from fractures has grown steadily, paving the way for therapeutic prevention and treatment of bone nonunion.

The early inflammatory response is the link between the immune system and bone tissue, which are coupled and controlled by one another during the complicated biological process of bone repair. The intrinsic immune response is triggered by injury or infection, and a significant number of immune cells enter the microenvironment to start the bone healing cascade.^34^ As the initial immune cells, neutrophils play a critical role in controlling acute inflammation as well as macrophage recruitment through the release of IL‐6 and CCL2. Through inter‐phenotypic changes, macrophages recruited in the microenvironment transition from neovascularization to maturation and from the early pro‐inflammatory state to the late healing stage. M1‐type macrophages regulate the release of cytokines by activated lymphocytes, which leads to an increase in chemokines and the recruitment of circulating neutrophils, aggravating the inflammatory response and impairing bone repair.^19^, ^43^, ^48^ The ongoing study of synergistic or antagonistic interactions between immune cells in the bone healing process is a promising area of research in the field of osteoimmunology.^17^, ^36^

In the study of the mechanism of fracture healing, the signaling pathways found to promote bone regeneration are mostly focused on the promotion of the proliferation of MSCs at the fracture breaks or the promotion of their osteogenic differentiation, and there are fewer studies on the cartilage spectrum. In addition, for the above immunoregulatory aspects, in addition to studies in *in vitro* systems such as cells, their roles need to be confirmed in different transgenic animal models and different fracture injury models. Transgenic animal models need to include conditional knockout models of different cellular lineages to corroborate the role of potential target molecules in different cells or at different stages of healing. Fracture injury modeling should also consider the species of the animal, the type of fracture, and the physiological condition of the animal, all of which can have an effect on fracture healing.

The gold standard for treating osteogenesis imperfecta is surgical grafting, but because of its complications, healing can still fail. As a result, it is important to continually find new and better ways to treat osteogenesis imperfecta, lessen patient discomfort, and keep treatment costs down. The creation and optimization of new grafting materials, such as the creation of finer nanoscale grafts in conjunction with three‐dimensional printing technology, as well as the creation of new composite materials to enhance osteogenic differentiation, angiogenesis, and bone regeneration, are current research hotspots in the field of bone grafting. Currently, a variety of immune cells and growth factors play crucial roles in the healing of fractures; however, the dose, manner, and frequency of growth factor delivery need to be continuously adjusted because they might have varying effects in different cells and at different phases of healing. To create more suitable local delivery systems, this method must be coupled with biotissue engineering.

## Conclusion

A series of studies on fracture healing have revealed the critical role of immune cells and their effector molecules in the healing process. In the article, we further revealed the mechanisms by which immune cells are recruited and activated, the effector molecules of immune cells, and the target cells of effector molecules. We found that in the early and late stages of fracture healing, the immune and inflammatory responses showed an early elevation, which induced enhanced osteogenic factors, and a significant decline in the late stage, whereas the strong immune and inflammatory responses in the late stage inhibited osteoblast formation. These findings will guide the regulation of immune mechanisms for future fracture healing and might provide better ideas for targeting therapies in the future development of bone replacement materials with immunomodulatory capabilities. The immune mechanisms are complex and variable, and we have not yet been able to clearly elucidate the mechanism of action of all the factors in the three phases of bone nonunion. Further, we lack clinical and experimental data. We will carry out experimental validation and mechanism analysis from key immune cells and factors in future studies.

## Author Contributions

This study was conceived and designed by Zongchao Liu. Chao Song wrote most of the manuscript. Yong Liu and Xingxing Tao were involved in the manuscript writing and revision process. Kang Cheng, Yang Zhou, Houyin Shi, and Liquan Wang collected the references and mapped figures. Weiye Cai, Daqian Zhou, and Qi Hao checked the manuscript. All authors listed have made a substantial contribution to the work.

## Conflict of Interest Statement

The authors have no financial or proprietary interests in any material discussed in this article.

## References

[1] Panteli M , Vun JSH , Pountos I , Howard A , Jones E , Giannoudis PV . Biological and molecular profile of fracture non‐union tissue: a systematic review and an update on current insights. J Cell Mol Med. 2022;26(3):601–623. 34984803 10.1111/jcmm.17096 PMC8817135

[2] Dozza B , Salamanna F , Baleani M , Giavaresi G , Parrilli A , Zani L , et al. Nonunion fracture healing: evaluation of effectiveness of demineralized bone matrix and mesenchymal stem cells in a novel sheep bone nonunion model. J Tissue Eng Regen Med. 2018;12(9):1972–1985. 30044550 10.1002/term.2732

[3] Megas P . Classification of non‐union. Injury. 2005;36(Suppl 4):S30–S37. 16291321 10.1016/j.injury.2005.10.008

[4] Hodgson H , Giannoudis PV , Howard A . Fracture non‐union; what are the current perceived challenges among clinicians? Injury. 2022;53(12):3865–3866. 36379739 10.1016/j.injury.2022.10.029

[5] Neumann MV , Zwingmann J , Jaeger M , Hammer TO , Südkamp NP . Non‐Union in Upper Limb Fractures—clinical evaluation and treatment options. Acta Chir Orthop Traumatol Cech. 2016;83(4):223–230. 28026722

[6] Pearson RG , Clement RG , Edwards KL , Scammell BE . Do smokers have greater risk of delayed and non‐union after fracture, osteotomy and arthrodesis? A systematic review with meta‐analysis. BMJ Open. 2016;6(11):e010303. 10.1136/bmjopen-2015-010303 PMC5129177 28186922

[7] van Basten BM , Houben IB , Blokhuis TJ . The non‐union scoring system: an interobserver reliability study. Eur J Trauma Emerg Surg. 2019;45(1):13–19. 28577203 10.1007/s00068-017-0796-4

[8] Lafuente‐Gracia L , Borgiani E , Nasello G , Geris L . Towards in silico models of the inflammatory response in bone fracture healing. Front Bioeng Biotechnol. 2021;9:703725. 34660547 10.3389/fbioe.2021.703725 PMC8514728

[9] Roberts SJ , Ke HZ . Anabolic strategies to augment bone fracture healing. Curr Osteoporos Rep. 2018;16(3):289–298. 29725836 10.1007/s11914-018-0440-1 PMC5945805

[10] Holmes D . Non‐union bone fracture: a quicker fix. Nature. 2017;550(7677):S193. 29069072 10.1038/550S193a

[11] Aro HT , Wippermann BW , Hodgson SF , Chao EY . Internal remodeling of periosteal new bone during fracture healing. J Orthop Res. 1990;8(2):238–246. 2303957 10.1002/jor.1100080213

[12] Kolar P , Schmidt‐Bleek K , Schell H , Gaber T , Toben D , Schmidmaier G , et al. The early fracture hematoma and its potential role in fracture healing. Tissue Eng, Part B. 2010;16(4):427–434. 10.1089/ten.TEB.2009.0687 20196645

[13] Hoff P , Gaber T , Strehl C , Jakstadt M , Hoff H , Schmidt‐Bleek K , et al. A pronounced inflammatory activity characterizes the early fracture healing phase in immunologically restricted patients. Int J Mol Sci. 2017;18(3):583. 10.3390/ijms18030583 PMC5372599 28282868

[14] Haffner‐Luntzer M , Heilmann A , Rapp AE , Beie S , Schinke T , Amling M , et al. Midkine‐deficiency delays chondrogenesis during the early phase of fracture healing in mice. PLoS One. 2014;9(12):e116282. 25551381 10.1371/journal.pone.0116282 PMC4281158

[15] Burska AN , Giannoudis PV , Tan BH , Ilas D , Jones E , Ponchel F . Dynamics of early Signalling events during fracture healing and potential serum biomarkers of fracture non‐Union in Humans. J Clin Med. 2020;9(2):492. 10.3390/jcm9020492 PMC7073571 32054088

[16] Thomson DD . Introduction—mechanisms of fracture healing and pharmacologic control. J Musculoskelet Neuronal Interact. 2003;3(4):295–296. 15758303

[17] Mountziaris PM , Mikos AG . Modulation of the inflammatory response for enhanced bone tissue regeneration. Tissue Eng Part B Rev. 2008;14(2):179–186. 18544015 10.1089/ten.teb.2008.0038 PMC2962857

[18] Geris L , Gerisch A , Sloten JV , Weiner R , Oosterwyck HV . Angiogenesis in bone fracture healing: a bioregulatory model. J Theor Biol. 2008;251(1):137–158. 18155732 10.1016/j.jtbi.2007.11.008

[19] Schlundt C , El Khassawna T , Serra A , Dienelt A , Wendler S , Schell H , et al. Macrophages in bone fracture healing: their essential role in endochondral ossification. Bone. 2018;106:78–89. 26529389 10.1016/j.bone.2015.10.019

[20] Goldhahn J , Féron JM , Kanis J , Papapoulos S , Reginster JY , Rizzoli R , et al. Implications for fracture healing of current and new osteoporosis treatments: an ESCEO consensus paper. Calcif Tissue Int. 2012;90(5):343–353. 22451221 10.1007/s00223-012-9587-4

[21] Tam WL , Freitas Mendes L , Chen X , Lesage R , Van Hoven I , Leysen E , et al. Human pluripotent stem cell‐derived cartilaginous organoids promote scaffold‐free healing of critical size long bone defects. Stem Cell Res Ther. 2021;12(1):513. 34563248 10.1186/s13287-021-02580-7 PMC8466996

[22] Hak DJ . Management of aseptic tibial nonunion. J Am Acad Orthop Surg. 2011;19(9):563–573. 21885702 10.5435/00124635-201109000-00007

[23] Faienza MF , Chiarito M , D'Amato G , Colaianni G , Colucci S , Grano M , et al. Monoclonal antibodies for treating osteoporosis. Expert Opin Biol Ther. 2018;18(2):149–157. 10.1080/14712598.2018.1401607 29113523

[24] Shapiro F , Wu JY . Woven bone overview: structural classification based on its integral role in developmental, repair and pathological bone formation throughout vertebrate groups. Eur Cell Mater. 2019;38:137–167. 31571191 10.22203/eCM.v038a11

[25] Schindeler A , McDonald MM , Bokko P , Little DG . Bone remodeling during fracture repair: the cellular picture. Semin Cell Dev Biol. 2008;19(5):459–466. 18692584 10.1016/j.semcdb.2008.07.004

[26] McKibbin B . The biology of fracture healing in long bones. J Bone Jt Surg Br. 1978;60‐b(2):150–162. 10.1302/0301-620X.60B2.350882 350882

[27] Wang M , Yang N , Wang X . A review of computational models of bone fracture healing. Med Biol Eng Comput. 2017;55(11):1895–1914. 28785849 10.1007/s11517-017-1701-3

[28] Khanna A , Gougoulias N , Maffulli N . Intermittent pneumatic compression in fracture and soft‐tissue injuries healing. Br Med Bull. 2008;88(1):147–156. 18596049 10.1093/bmb/ldn024

[29] Wang H , Wei X , Liu P , Fu YH , Wang PF , Cong YX , et al. Quality of life and complications at the different stages of bone transport for treatment infected nonunion of the tibia. Medicine. 2017;96(45):e8569. 29137077 10.1097/MD.0000000000008569 PMC5690770

[30] de Seny D , Cobraiville G , Leprince P , Fillet M , Collin C , Mathieu M , et al. Biomarkers of inflammation and innate immunity in atrophic nonunion fracture. J Transl Med. 2016;14(1):258. 27599571 10.1186/s12967-016-1019-1 PMC5011805

[31] Horton JE , Raisz LG , Simmons HA , Oppenheim JJ , Mergenhagen SE . Bone resorbing activity in supernatant fluid from cultured human peripheral blood leukocytes. Science. 1972;177(4051):793–795. 5052733 10.1126/science.177.4051.793

[32] Tsukasaki M , Takayanagi H . Osteoimmunology: evolving concepts in bone‐immune interactions in health and disease. Nat Rev Immunol. 2019;19(10):626–642. 31186549 10.1038/s41577-019-0178-8

[33] Okamoto K , Takayanagi H . Osteoimmunology. Cold Spring Harb Perspect Med. 2019;9:1. 10.1101/cshperspect.a031245 PMC6314075 29610150

[34] Song C , Cai W , Liu F , Cheng K , Guo D , Liu Z . An in‐depth analysis of the immunomodulatory mechanisms of intervertebral disc degeneration. JOR Spine. 2022;5(4):e1233. 36601372 10.1002/jsp2.1233 PMC9799087

[35] Song C , Zhou Y , Cheng K , Liu F , Cai W , Zhou D , et al. Cellular senescence—molecular mechanisms of intervertebral disc degeneration from an immune perspective. Biomed Pharmacother. 2023;162:114711. 37084562 10.1016/j.biopha.2023.114711

[36] Maruyama M , Rhee C , Utsunomiya T , Zhang N , Ueno M , Yao Z , et al. Modulation of the inflammatory response and bone healing. Front Endocrinol. 2020;11:386. 10.3389/fendo.2020.00386 PMC7325942 32655495

[37] Qin Y , Guan J , Zhang C . Mesenchymal stem cells: mechanisms and role in bone regeneration. Postgrad Med J. 2014;90(1069):643–647. 25335795 10.1136/postgradmedj-2013-132387 PMC4215372

[38] Kapasa ER , Giannoudis PV , Jia X , Hatton PV , Yang XB . The effect of RANKL/OPG balance on reducing implant complications. J Funct Biomater. 2017;8(4):42. 10.3390/jfb8040042 PMC5748549 28937598

[39] Xiong Y , Mi BB , Lin Z , Hu YQ , Yu L , Zha KK , et al. The role of the immune microenvironment in bone, cartilage, and soft tissue regeneration: from mechanism to therapeutic opportunity. Mil Med Res. 2022;9(1):65. 36401295 10.1186/s40779-022-00426-8 PMC9675067

[40] Pajarinen J , Lin T , Gibon E , Kohno Y , Maruyama M , Nathan K , et al. Mesenchymal stem cell‐macrophage crosstalk and bone healing. Biomaterials. 2019;196:80–89. 29329642 10.1016/j.biomaterials.2017.12.025 PMC6028312

[41] Loi F , Córdova LA , Zhang R , Pajarinen J , Lin TH , Goodman SB , et al. The effects of immunomodulation by macrophage subsets on osteogenesis in vitro. Stem Cell Res Ther. 2016;7:15. 26801095 10.1186/s13287-016-0276-5 PMC4724110

[42] Gibon E , Loi F , Córdova LA , Pajarinen J , Lin T , Lu L , et al. Aging affects bone marrow macrophage polarization: relevance to bone healing. Regen Eng Transl Med. 2016;2(2):98–104. 28138512 10.1007/s40883-016-0016-5 PMC5270653

[43] Freytes DO , Kang JW , Marcos‐Campos I , Vunjak‐Novakovic G . Macrophages modulate the viability and growth of human mesenchymal stem cells. J Cell Biochem. 2013;114(1):220–229. 22903635 10.1002/jcb.24357

[44] Chang MK , Raggatt LJ , Alexander KA , Kuliwaba JS , Fazzalari NL , Schroder K , et al. Osteal tissue macrophages are intercalated throughout human and mouse bone lining tissues and regulate osteoblast function in vitro and in vivo. J Immunol. 2008;181(2):1232–1244. 18606677 10.4049/jimmunol.181.2.1232

[45] Vi L , Baht GS , Whetstone H , Ng A , Wei Q , Poon R , et al. Macrophages promote osteoblastic differentiation in‐vivo: implications in fracture repair and bone homeostasis. J Bone Miner Res. 2015;30(6):1090–1102. 25487241 10.1002/jbmr.2422

[46] Schlundt C , Fischer H , Bucher CH , Rendenbach C , Duda GN , Schmidt‐Bleek K . The multifaceted roles of macrophages in bone regeneration: a story of polarization, activation and time. Acta Biomater. 2021;133:46–57. 33974949 10.1016/j.actbio.2021.04.052

[47] Duda GN , Geissler S , Checa S , Tsitsilonis S , Petersen A , Schmidt‐Bleek K . The decisive early phase of bone regeneration. Nat Rev Rheumatol. 2023;19(2):78–95. 36624263 10.1038/s41584-022-00887-0

[48] Snyder RJ , Lantis J , Kirsner RS , Shah V , Molyneaux M , Carter MJ . Macrophages: a review of their role in wound healing and their therapeutic use. Wound Repair Regen. 2016;24(4):613–629. 27196106 10.1111/wrr.12444

[49] Yang N , Liu Y . The role of the immune microenvironment in bone regeneration. Int J Med Sci. 2021;18(16):3697–3707. 34790042 10.7150/ijms.61080 PMC8579305

[50] Zhao Z , Hou X , Yin X , Li Y , Duan R , Boyce BF , et al. TNF induction of NF‐κB RelB enhances RANKL‐induced Osteoclastogenesis by promoting inflammatory macrophage differentiation but also limits it through suppression of NFATc1 expression. PLoS One. 2015;10(8):e0135728. 26287732 10.1371/journal.pone.0135728 PMC4545392

[51] Berardi S , Corrado A , Maruotti N , Cici D , Cantatore FP . Osteoblast role in the pathogenesis of rheumatoid arthritis. Mol Biol Rep. 2021;48(3):2843–2852. 33774802 10.1007/s11033-021-06288-y PMC8060181

[52] Wustrow TP . Biology of interleukin‐1 (IL‐1), with respect to otorhinolaryngology‐head and neck surgery. Head Neck. 1994;16(1):88–94. 8125795 10.1002/hed.2880160118

[53] McKee MD , Pedraza CE , Kaartinen MT . Osteopontin and wound healing in bone. Cells Tissues Organs. 2011;194(2–4):313–319. 21576907 10.1159/000324244

[54] Ono T , Takayanagi H . Osteoimmunology in bone fracture healing. Curr Osteoporos Rep. 2017;15(4):367–375. 28647888 10.1007/s11914-017-0381-0

[55] Groom JR . Moving to the suburbs: T‐cell positioning within lymph nodes during activation and memory. Immunol Cell Biol. 2015;93(4):330–336. 25753266 10.1038/icb.2015.29

[56] Miyajima A , Tanaka M , Itoh T . Stem/progenitor cells in liver development, homeostasis, regeneration, and reprogramming. Cell Stem Cell. 2014;14(5):561–574. 24792114 10.1016/j.stem.2014.04.010

[57] Li J , Tan J , Martino MM , Lui KO . Regulatory T‐cells: potential regulator of tissue repair and regeneration. Front Immunol. 2018;9:585. 29662491 10.3389/fimmu.2018.00585 PMC5890151

[58] Colburn NT , Zaal KJ , Wang F , Tuan RS . A role for gamma/delta T cells in a mouse model of fracture healing. Arthritis Rheum. 2009;60(6):1694–1703. 19479830 10.1002/art.24520 PMC2697263

[59] Aurora R , Silva MJ . T cells heal bone fractures with help from the gut microbiome. J Clin Invest. 2023;133(8):e167311. 10.1172/JCI167311 PMC10104886 37066879

[60] Hu Y , Cui Q , Luo C , Luo Y , Shi J , Huang H . A promising sword of tomorrow: human γδ T cell strategies reconcile allo‐HSCT complications. Blood Rev. 2016;30(3):179–188. 26654098 10.1016/j.blre.2015.11.002

[61] Dixit M , Singh KB , Prakash R , Singh D . Functional block of IL‐17 cytokine promotes bone healing by augmenting FOXO1 and ATF4 activity in cortical bone defect model. Osteoporos Int. 2017;28(7):2207–2220. 28341898 10.1007/s00198-017-4012-5

[62] Pacifici R . The role of IL‐17 and TH17 cells in the bone catabolic activity of PTH. Front Immunol. 2016;7:57. 26925062 10.3389/fimmu.2016.00057 PMC4756106

[63] Fei Y , Gronowicz G , Hurley MM . Fibroblast growth factor‐2, bone homeostasis and fracture repair. Curr Pharm Des. 2013;19(19):3354–3363. 23432676 10.2174/1381612811319190002

[64] Guo YC , Yuan Q . Fibroblast growth factor 23 and bone mineralisation. Int J Oral Sci. 2015;7(1):8–13. 25655009 10.1038/ijos.2015.1 PMC4817534

[65] Bollenbecker S , Barnes JW , Krick S . Fibroblast growth factor signaling in development and disease. Int J Mol Sci. 2023;24(11):9734. 10.3390/ijms24119734 PMC10253427 37298683

[66] Le Blanc S , Simann M , Jakob F , Schütze N , Schilling T . Fibroblast growth factors 1 and 2 inhibit adipogenesis of human bone marrow stromal cells in 3D collagen gels. Exp Cell Res. 2015;338(2):136–148. 26384550 10.1016/j.yexcr.2015.09.009

[67] Wang J , Liu S , Li J , Yi Z . The role of the fibroblast growth factor family in bone‐related diseases. Chem Biol Drug Des. 2019;94(4):1740–1749. 31260189 10.1111/cbdd.13588

[68] Hurley MM , Adams DJ , Wang L , Jiang X , Burt PM , Du E , et al. Accelerated fracture healing in transgenic mice overexpressing an anabolic isoform of fibroblast growth factor 2. J Cell Biochem. 2016;117(3):599–611. 26252425 10.1002/jcb.25308

[69] Bosetti M , Leigheb M , Brooks RA , Boccafoschi F , Cannas MF . Regulation of osteoblast and osteoclast functions by FGF‐6. J Cell Physiol. 2010;225(2):466–471. 20458746 10.1002/jcp.22225

[70] Xu J , Huang Z , Wang W , Tan X , Li H , Zhang Y , et al. FGF8 signaling alters the osteogenic cell fate in the hard palate. J Dent Res. 2018;97(5):589–596. 29342370 10.1177/0022034517750141 PMC5958370

[71] Hung IH , Schoenwolf GC , Lewandoski M , Ornitz DM . A combined series of Fgf9 and Fgf18 mutant alleles identifies unique and redundant roles in skeletal development. Dev Biol. 2016;411(1):72–84. 26794256 10.1016/j.ydbio.2016.01.008 PMC4801039

[72] Wang L , Roth T , Abbott M , Ho L , Wattanachanya L , Nissenson RA . Osteoblast‐derived FGF9 regulates skeletal homeostasis. Bone. 2017;98:18–25. 28189801 10.1016/j.bone.2016.12.005 PMC8474898

[73] Schmid GJ , Kobayashi C , Sandell LJ , Ornitz DM . Fibroblast growth factor expression during skeletal fracture healing in mice. Dev Dyn. 2009;238(3):766–774. 19235733 10.1002/dvdy.21882 PMC2688661

[74] Guimarães JM , Guimarães IC , Duarte ME , Vieira T , Vianna VF , Fernandes MB , et al. Polymorphisms in BMP4 and FGFR1 genes are associated with fracture non‐union. J Orthop Res. 2013;31(12):1971–1979. 23939983 10.1002/jor.22455

[75] Xu W , Luo F , Wang Q , Tan Q , Huang J , Zhou S , et al. Inducible activation of FGFR2 in adult mice promotes bone formation after bone marrow ablation. J Bone Miner Res. 2017;32(11):2194–2206. 28650109 10.1002/jbmr.3204

[76] Chen H , Sun X , Yin L , Chen S , Zhu Y , Huang J , et al. PTH 1‐34 ameliorates the osteopenia and delayed healing of stabilized tibia fracture in mice with achondroplasia resulting from gain‐of‐function mutation of FGFR3. Int J Biol Sci. 2017;13(10):1254–1265. 29104492 10.7150/ijbs.21258 PMC5666524

[77] Su N , Yang J , Xie Y , Du X , Lu X , Yin Z , et al. Gain‐of‐function mutation of FGFR3 results in impaired fracture healing due to inhibition of chondrocyte differentiation. Biochem Biophys Res Commun. 2008;376(3):454–459. 18789890 10.1016/j.bbrc.2008.08.165

[78] Su N , Li X , Tang Y , Yang J , Wen X , Guo J , et al. Deletion of FGFR3 in osteoclast lineage cells results in increased bone mass in mice by inhibiting Osteoclastic bone resorption. J Bone Miner Res. 2016;31(9):1676–1687. 26990430 10.1002/jbmr.2839

[79] Bai J , Ge G , Wang Q , Li W , Zheng K , Xu Y , et al. Engineering stem cell recruitment and Osteoinduction via bioadhesive molecular mimics to improve osteoporotic bone‐implant integration. Research. 2022;2022:9823784. 36157511 10.34133/2022/9823784 PMC9484833

[80] Carreira AC , Lojudice FH , Halcsik E , Navarro RD , Sogayar MC , Granjeiro JM . Bone morphogenetic proteins: facts, challenges, and future perspectives. J Dent Res. 2014;93(4):335–345. 24389809 10.1177/0022034513518561

[81] Van Lieshout EMM , Den Hartog D . Effect of platelet‐rich plasma on fracture healing. Injury. 2021;52(Suppl 2):S58–s66. 10.1016/j.injury.2020.12.005 33431160

[82] Baldwin P , Li DJ , Auston DA , Mir HS , Yoon RS , Koval KJ . Autograft, allograft, and bone graft substitutes: clinical evidence and indications for use in the setting of Orthopaedic trauma surgery. J Orthop Trauma. 2019;33(4):203–213. 30633080 10.1097/BOT.0000000000001420

[83] Madison RD , Nowotarski PJ . The reamer‐irrigator‐aspirator in nonunion surgery. Orthop Clin North Am. 2019;50(3):297–304. 31084831 10.1016/j.ocl.2019.03.001

[84] Su JC , Liu XW , Yu BQ , Li ZD , Li M , Zhang CC . Shape memory Ni‐Ti alloy swan‐like bone connector for treatment of humeral shaft nonunion. Int Orthop. 2010;34(3):369–375. 19198838 10.1007/s00264-009-0726-0 PMC2899287

[85] Dreyer CH , Jørgensen NR , Overgaard S , Qin L , Ding M . Vascular endothelial growth factor and mesenchymal stem cells revealed similar bone formation to allograft in a sheep model. Biomed Res Int. 2021;2021:6676609. 33763484 10.1155/2021/6676609 PMC7946458

[86] Yang X , Jiang T , Wang Y , Guo L . The role and mechanism of SIRT1 in resveratrol‐regulated osteoblast autophagy in osteoporosis rats. Sci Rep. 2019;9(1):18424. 31804494 10.1038/s41598-019-44766-3 PMC6895060

[87] Liang G , Zhao J , Pan J , Yang Y , Dou Y , Yang W , et al. Network pharmacology identifies fisetin as a treatment for osteoporosis that activates the Wnt/β‐catenin signaling pathway in BMSCs. J Orthop Surg Res. 2023;18(1):312. 37087476 10.1186/s13018-023-03761-1 PMC10122799

[88] Murahashi Y , Yano F , Nakamoto H , Maenohara Y , Iba K , Yamashita T , et al. Multi‐layered PLLA‐nanosheets loaded with FGF‐2 induce robust bone regeneration with controlled release in critical‐sized mouse femoral defects. Acta Biomater. 2019;85:172–179. 30583110 10.1016/j.actbio.2018.12.031

[89] Baht GS , Vi L , Alman BA . The role of the immune cells in fracture healing. Curr Osteoporos Rep. 2018;16(2):138–145. 29508143 10.1007/s11914-018-0423-2 PMC5866272

